# The complete mitochondrial genome of a rarely reported porcelain crab, *Pisidia striata* (Anomura, Galatheoidea, Porcellanidae), from the Chinese waters

**DOI:** 10.1080/23802359.2025.2467162

**Published:** 2025-02-20

**Authors:** Xifei Wang, Xuefeng Fang, Dong Dong

**Affiliations:** ^a^ College of Life Sciences, Qingdao Agricultural University, Qingdao, China; ^b^ Department of Marine Organism Taxonomy & Phylogeny, Qingdao Key Laboratory of Marine Biodiversity and Conservation, Institute of Oceanology, Chinese Academy of Sciences, Qingdao, China; ^c^ University of Chinese Academy of Sciences, Beijing, China

**Keywords:** Mitogenome, Porcellanidae, phylogeny

## Abstract

The species *Pisidia striata* is predominantly distributed along the southeastern coast of China and belongs to the shallow-water crab family Porcellanidae. The complete mitochondrial genome of *P. striata*, which spans 15,357 bp and contains 13 protein-coding genes, 22 tRNAs and 2 rRNAs, is presented in this study. The nucleotide composition reveals A (37.81%), T (35.48%), G (9.75%), and C (16.96%). Phylogenetic analysis demonstrates a close relationship between *P. striata* and *P. serratifrons* within the *Pisidia* clade with robust bootstrap support values. This mitochondrial genome will be a significant supplement for the genus *Pisidia* and whole mitogenome phylogenetic analysis provided insights into further evolutionary research of Porcellanidae.

## Introduction

*Pisidia striata* Yang and Sun 1990 inhabits low-tidal regions, either beneath rocks or on coarse-grained sand substrates. It is also observed hiding inside the oyster and scallop cages (Yang and Sun 1990). Currently, it is only known to inhabit Chinese waters, with its distribution range extending from Shandong Province in northern China to Fujian Province in southeastern China. Morphologically similar to *Pisidia serratifrons* (Stimpson 1858), *P. striata* possesses a broad median rostral lobe that is anteriorly serrated and nearly twice as wide as the lateral lobes. Both species are sympatric in China and can be easily mistaken for each other. *Pisidia striata* can be distinguished from the latter by having the anterior margins of the rostral lobes weakly serrated instead of distinctly spinose or denticulate; the dorsal surface of the smaller chela rugose along the midline ridge, instead of crenulated or tuberculate; and the carpus of the cheliped only with distal spine on the dorso-extensor margin instead of armed with two spines. Unlike its relative *P. serratifrons*, there have been limited reports on *P. striata* since its establishment, resulting in a lack of comprehensive molecular genetics studies (Yang and Sun 1990). The genus *Pisidia* Leach 1821 currently comprises 14 species worldwide (Osawa and McLaughlin 2010), with only one species, *P. serratifrons*, has been sequenced for the mitochondrial genome (Lü et al. 2023).

In this study, we provided the complete mitochondrial genome of *P. striata*, which represents the second mitochondrial genome of this genus and the fourth within the family Porcellanidae. This research contributes to a better understanding of macrobenthos biodiversity in Chinese coastal waters and enhances the available mitochondrial molecular data for further phylogenetic investigations within Porcellanidae.

## Materials and methods

**Figure 1.**
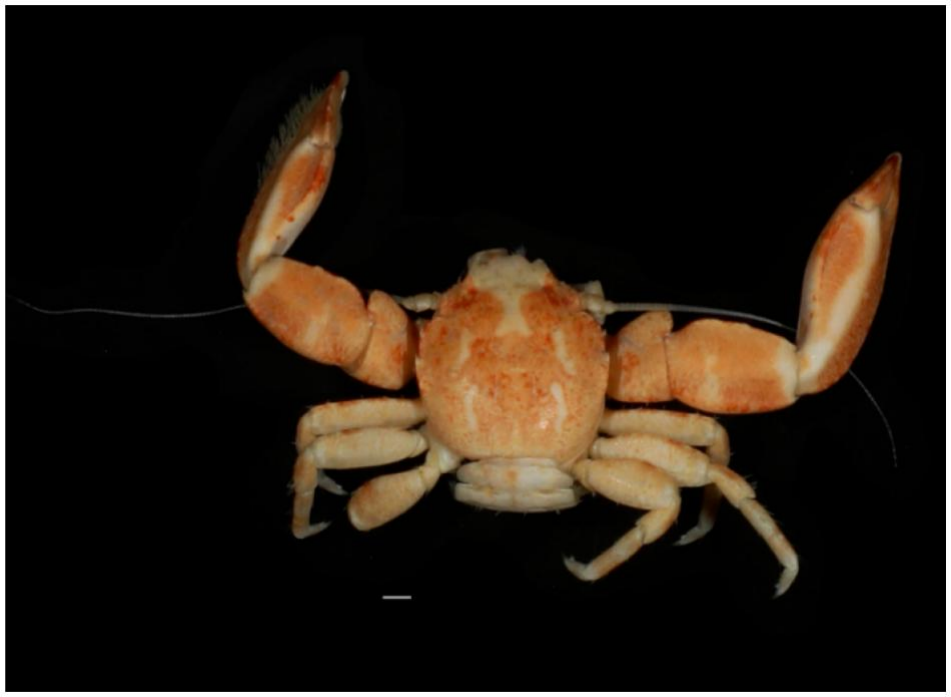
Fresh photo of *P. striata* that taken by Dong Dong, MBM189318. Scale equals is 1.0 mm.

The *P. striata* samples were collected from the subtidal zone in their living states at two sites: Dingzi Bay (36°38’N, 121°08’E), Shandong Province, in June 2022 and Haizhou Bay (35°01’N, 119°17’E), Jiangsu Province, in April 2024 (Figure 1). The specimens are deposited at the Marine Biological Museum (Dong Dong, dongd@qdio.ac.cn), Institute of Oceanology, Chinese Academy of Sciences, under the voucher numbers: MBM189317 (Dingzi Bay) and MBM189318 (Haizhou Bay). A specimen in MBM189317 was selected for the mitogenome sequencing. Whole genome DNA was extracted using the E.Z.N.A^®^ Tissue DNAkit (OMEGA). The DNA sample was sent to Biozeron Co. Ltd (Shanghai, China) for whole-genome sequencing using the Illumina TruSeq^™^ Nano DNA Sample Prep Kit method to construct the library. Subsequently, the library underwent 2×150 bp sequencing on the Illumina NovaSeq platform, resulting in a total of 80,973,172 clean reads obtained through Illumina HiSeq sequencing. The mitochondrial genome of *P. striata* was assembled using GetOrganelle v1.7.5 (Jin et al. 2020), and then annotated *via* the MITOS webserve (http://mitos.bioinf.uni-leipzig.de/index.py) (Bernt et al. 2013). Finally, the initiator/terminator condons of each protein-coding gene (PCG) were manually corrected in SnapGene Viewer with reference to the mitochondrial genome of *P. serratifrons* (GenBank: OM461359) (Lü et al. 2023).

For the phylogenetic study, 13 PCGs from *P. striata* and nine other galatheoid species belonging to four different lineages – Porcellanidae, Munididae, Munidopsidae and Galatheidae – were analyzed (Supplemental Table S1). Two hermit crabs, *Dardanus aspersus* (Berthold, 1846) and *D. arrosor* (Herbst, 1796), were selected as the outgroups. The nucleotide sequences of each gene were aligned and trimmed using MEGA 6.0 (Tamura et al. 2013). Subsequently, the 13 PCGs were concatenated using PhyloSuite (Zhang et al. 2020). The phylogenetic relationships, based on the concatenated sequences, were inferred employing the maximum likelihood (ML) method implemented in the IQ-TREE (Nguyen et al. 2015). The best nucleotide base substitution model for each gene selected in present study was determined by PartitionFinder 2 (Lanfear et al. 2017) under the Akaike Information Criterion (AIC). The implementation of PartitionFinder 2 and IQ-TREE were pipelined in the program PhyloSuite. To validate the robustness of tree topology obtained, nodal support was evaluated with 5000 ultrafast bootstrap replicates (Minh et al. 2013). Eventually, the final phylogenetic tree visualization was accomplished using iTOL (Letunic and Bork 2019).

## Results

**Figure 2.**
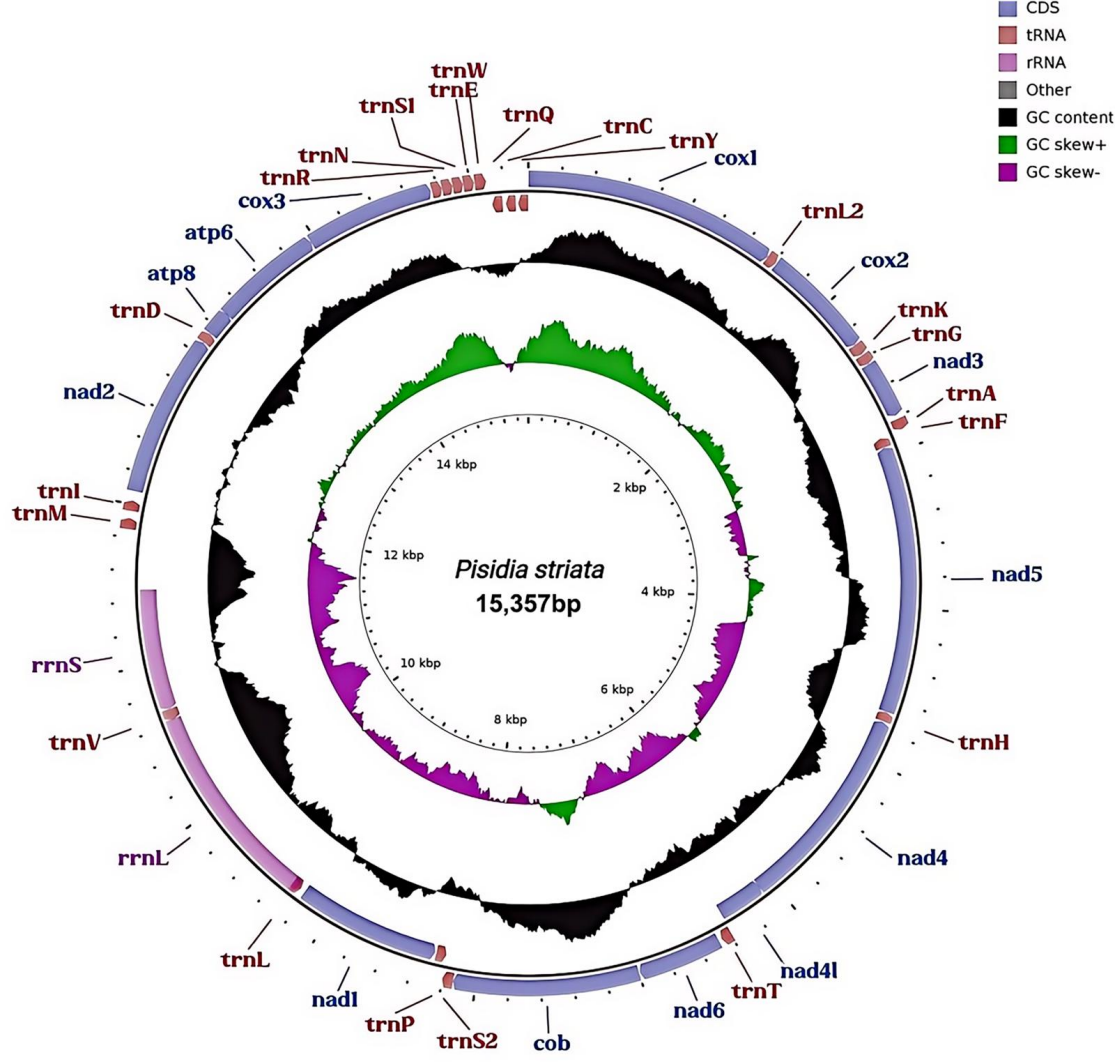
The organization of the mitogenome of *P. striata*. Genes for proteins and rRNAs are shown with standard abbreviations. Genes for tRNAs are represented by a single letter for the corresponding amino acid, with two leucine tRNAs and two serine tRNAs differentiated by numerals.

The assembled mitochondrial genome displayed a range of read mapping depths, with the minimum depth being 7× and the maximum depth reaching 3442×. The average depth was calculated to be 1025.86× (Supplementary Figure 1). The complete mitochondrial genome of *P. striata* is 15,357 bp in length, encompassing a total of 13 PCGs, 22 tRNAs and two rRNAs (Figure 2). Among these genes, 23 were transcribed on the heavy (H) strand, while four PCGs (i.e. *nad5*, *nad4*, *nad4l* and *nad1*), eight tRNAs (i.e. *trnF*, *trnH*, *trnP*, *trnL1*, *trnV*, *trnQ*, *trnC* and *trnY*), as well as two rRNAs (*rrnL* and *rrnS*) were located on the light (L-) strand. The *P. striata* mitogenome exhibited a total of six overlapping regions, including *rrnL* (50 bp), *nad4L*/*atp6* (7 bp), *cox3* (1 bp), *trnS2* (2 bp), and *trnV* (6 bp). Additionally, it contained 637 bp of intergenic spacers distributed across 19 regions, indicating the occurrence of tandem duplications and redundant gene deletions. The nucleotide composition of the mitogenome displayed a significant bias toward AT content (A: 37.81%, T: 35.48%, G: 9.75%, C: 16.96%). Genomic AT skewness was calculated as 0.270, while GC skewness was determined to be 0.032. All PCGs were initiated by an initiator codon ATN (ATT, ATG, ATA), except for cob which utilized TTG as its initiation codon. Regarding termination codons usage, *cox1* and *cox2* terminated with an incomplete termination codon (T -), whereas all other PCGs terminated with TAA codons. The lengths of the two ribosomal RNA genes (*rrnS* and *rrnL*) were measured at 781 bp and 1370 bp, respectively. A total of twenty-two tRNAs ranging in length from 63 bp (*trnR*) to 74 bp (*trnV*) demonstrated a substantial skewness toward high AT content at approximately 75.47%.

**Figure 3.**
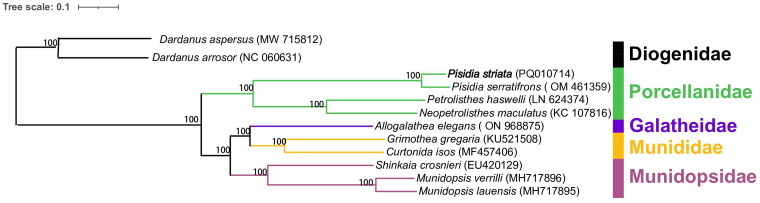
The maximum-likelihood (ML) phylogenetic tree for *P. striata* and the other Galatheoidea species based on the concatenated nucleotide sequences of 13 mitochondrial protein-coding genes. Bootstrap support values are indicated at each node. All species in the tree are labeled with their scientific names and GenBank accession numbers on the right side. The position of *P. striata* is bold fonts. A list of scientific names, accession numbers, and references for all sequences used in this phylogeny is provided in Supplemental Table S1.

Based on the phylogenetic tree, the *P. striata* belongs to the clade of Porcellanidae and is most closely related to *P. serratifrons* (Figure 3).

## Discussion and conclusion

The *P. striata* mitogenome was subjected to sequencing analysis in this study, leading to the acquisition of new mitochondrial data for Porcellanidae. Our investigation covered various features of *P. striata*, including AT-skew and codon usage bias. The G content of *P. striata* is 9.75%, which is similar to its closely related conspecific, *P. serratifrons* (Lü et al. 2023), showing a relatively low proportion within the family Porcellanidae. In contrast, the G contents of the *Petrolisthes haswelli* and *Neopetrolisthes maculatus* were recorded as 11.34% and 11.35%, respectively (Shen et al. 2013; Tan et al. 2016). The phylogenetic relationships among the four Galatheoidea lineages were consistent with previous studies (Tan et al. 2018; Palero et al. 2019). Furthermore, this study provides valuable mitogenomic data for future investigations into the evolutionary history of Galatheoidea.

## Supplementary Material

Table S1.docx

Supplementary Figure1.pdf

## Data Availability

The genome sequence data that support the findings of this study are openly available in GenBank of NCBI at https://www.ncbi.nlm.nih.gov under the accession no. PQ010714. The associated Bio-Sample, BioProject, and SRA numbers are SAMN43309092, PRJNA1151262 and SRR30350838, respectively.
