# Supplementary material for: The complete mitochondrial genome of a rarely reported porcelain crab, Pisidia striata (Anomura, Galatheoidea, Porcellanidae), from the Chinese waters
Source: Mitochondrial DNA B Resour. 2025 Feb 20;10(3):244–7. doi: 10.1080/23802359.2025.2467162 (PMC11843626; doi:10.1080/23802359.2025.2467162)
Supplement: Table S1.docx [file TMDN_A_2467162_SM9186.docx]

Table S1. Species and GenBank accesion numbers of sequences included in present study.

| Species | GenBank | References |
| --- | --- | --- |
| *Dardanus aspersus* | MW715812 | Lu (unpublished) |
| *Dardanus arrosor* | NC 060631 | Zhang et al. (2021) |
| *Pisidia serratifrons* | OM461359 | Lü et al. (2023) |
| *Petrolisthes haswelli* | LN624374 | Tan et al. (2014) |
| *Neopetrolisthes maculatus* | KC107816 | Shen et al. (2012) |
| *Allogalathea elegans* | ON968875 | Ruan et al. (2012) |
| *Grimothea gregaria* | KU521508 | Lee et al. (2016) |
| *Curtonida isos* | MF457406 | Tan et al. (2018) |
| *Shinkaia crosnieri* | EU420129 | Yang et al. (2008) |
| *Munidopsis verrilli* | MH717896 | Sun et al. (2019) |
| *Munidopsis lauensis* | MH717895 | Sun et al. (2019) |

**References**

Lee CW, Song JH, Min GS, Kim S. 2016. The complete mitochondrial genome of squat lobster, *Munida gregaria* (Anomura, Galatheoidea, Munididae). Mitochondrial DNA Part B. 1(1):204-206.

Lü J, Dong X, Li J, Ye Y, Xu K. 2023. Novel gene re-arrangement in the mitochondrial genome of *Pisidia serratifrons* (Anomura, Galatheoidea, Porcellanidae) and phylogenetic associations in Anomura. Biodivers Data J. 11: e96231.

Ruan X, Cheng H, Xuan Z, Li Z, Yu J, Zhao H. 2023. The complete mitochondrial genome of *Allogalathea elegans* (Adams & White, 1848) (Decapoda: Galatheidae). Mitochondrial DNA Part B. 8(8):857-861.

Shen H, Braband A, Scholtz G. 2013. Mitogenomic analysis of decapod crustacean phylogeny corroborates traditional views on their relationships. Mol Phylogenet Evol. 66(3):776-89.

Sun S, Sha Z, Wang Y. 2019. The complete mitochondrial genomes of two vent squat lobsters, *Munidopsis lauensis* and *M. verrilli*: Novel gene arrangements and phylogenetic implications. Ecol Evol. 9(22):12390-12407.

Tan MH, Gan HM, Lee YP, Austin CM. 2016. The complete mitogenome of the porcelain crab *Petrolisthes haswelli* Miers, 1884 (Crustacea: Decapoda: Anomura). Mitochondrial DNA Part A. 27(6):3983-3984.

Tan MH, Gan HM, Lee YP, Linton S, Grandjean F, Bartholomei-Santos ML, Miller AD, Austin CM. 2018. ORDER within the chaos: Insights into phylogenetic relationships within the Anomura (Crustacea: Decapoda) from mitochondrial sequences and gene order rearrangements. Mol Phylogenet Evol. 127:320-331.

Yang JS, Nagasawa H, Fujiwara Y, Tsuchida S, Yang WJ. 2008. The complete mitochondrial genome sequence of the hydrothermal vent galatheid crab *Shinkaia crosnieri* (Crustacea: Decapoda: Anomura): a novel arrangement and incomplete tRNA suite. BMC Genomics. 9:257.

Zhang Y, Meng L, Wei L, Lu X, Liu B, Liu L, Lü Z, Gao Y, Gong L. 2021. Different gene rearrangements of the genus *Dardanus* (Anomura: Diogenidae) and insights into the phylogeny of Paguroidea. Sci Rep. 11(1):21833.
