# Supplementary material for: The complete mitochondrial genome of a rarely reported porcelain crab, Pisidia striata (Anomura, Galatheoidea, Porcellanidae), from the Chinese waters
Source: Mitochondrial DNA B Resour. 2025 Feb 20;10(3):244–7. doi: 10.1080/23802359.2025.2467162 (PMC11843626; doi:10.1080/23802359.2025.2467162)
Supplement: Supplementary Figure1.pdf [file TMDN_A_2467162_SM9185.pdf]

Sequencing Depth and Coverage Map

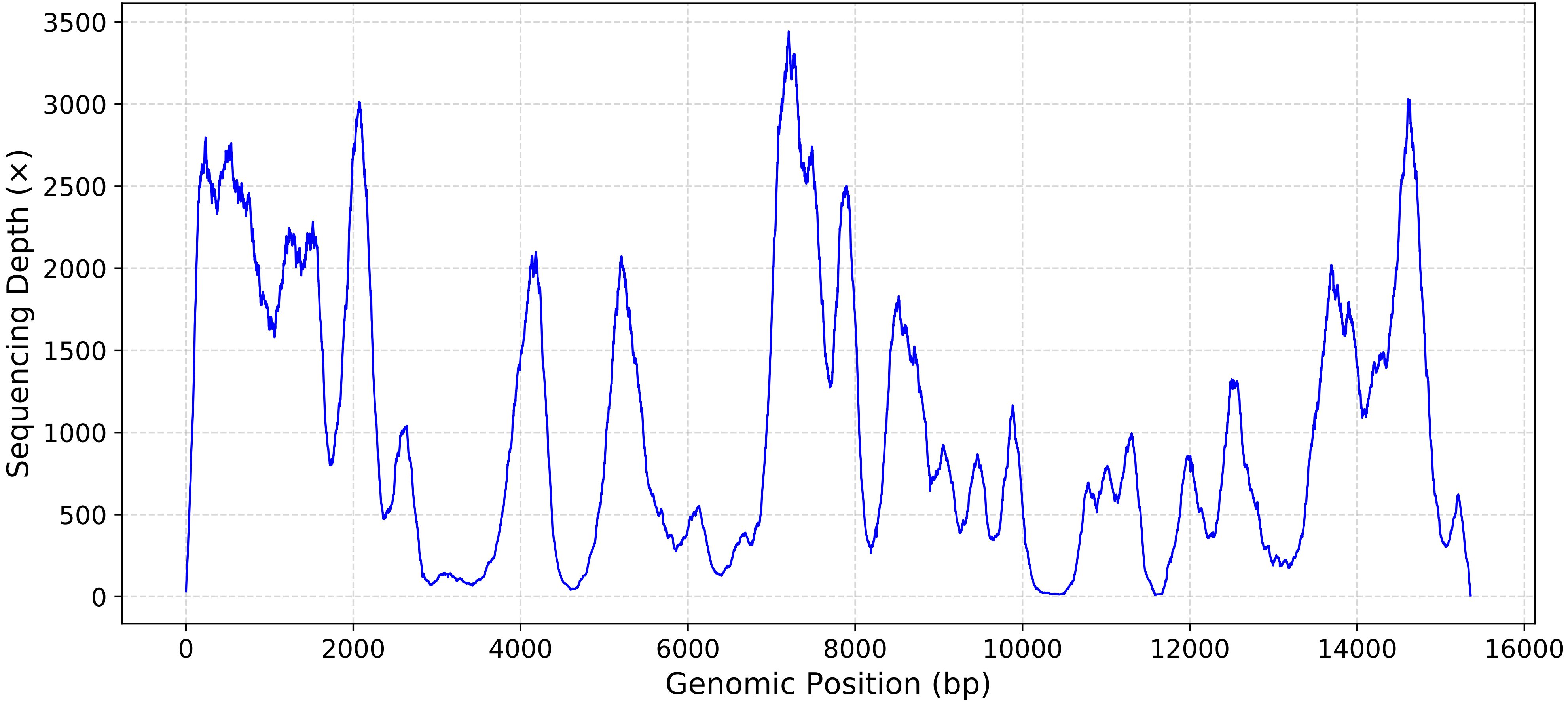

(1) Total genome length = 15,357 bp
(2) Average depth = 1025.86 ×
(3) Maximal depth = 3442 ×
(4) Minimal depth = 7 ×
